# Sustainable Carbon as Efficient Support for Metal-Based Nanocatalyst: Applications in Energy Harvesting and Storage

**DOI:** 10.3390/molecules25143123

**Published:** 2020-07-08

**Authors:** Mireia Buaki-Sogó, Leire Zubizarreta, Marta García-Pellicer, Alfredo Quijano-López

**Affiliations:** 1 Instituto Tecnológico de la Energia (ITE), Av. Juan de la Cierva 24, 46980 Valencia, Spain; marta.garcia@ite.es; 2 Universitat Politècnica de València, Camino de Vera s/n Edificio 6C, 46022 Valencia, Spain; alfredo.quijano@ite.es

**Keywords:** biomass, biochar, metal nanocatalyst, methanation reaction, sustainable carbon, energy storage

## Abstract

Sustainable activated carbon can be obtained from the pyrolysis/activation of biomass wastes coming from different origins. Carbon obtained in this way shows interesting properties, such as high surface area, electrical conductivity, thermal and chemical stability, and porosity. These characteristics among others, such as a tailored pore size distribution and the possibility of functionalization, lead to an increased use of activated carbons in catalysis. The use of activated carbons from biomass origins is a step forward in the development of more sustainable processes enhancing material recycling and reuse in the frame of a circular economy. In this article, a perspective of different heterogeneous catalysts based on sustainable activated carbon from biomass origins will be analyzed focusing on their properties and catalytic performance for determined energy-related applications. In this way, the article aims to give the reader a scope of the potential of these tailor-made sustainable materials as a support in heterogeneous catalysis and future developments needed to improve catalyst performance. The selected applications are those related with H_2_ energy and the production of biomethane for energy through CO_2_ methanation.

## 1. Introduction

The growth of the global population, exterior energy dependence, price instability, resource depletion, and environmental concern are some of the key factors that have led the EU to establish new policies. These policies are based on bio-economy and on European Commission strategies with ambitious objectives for 2020, such as: Reducing greenhouse gas emissions by 20%, increasing process energy efficiency by 20%, and reaching 20% in renewable energy consumption [1]. In this sense, the European Commission has been working on the law-making process in order to boost the use of renewable energies and every member state is obliged to elaborate a roadmap to achieve the objectives determined by these new European directives.

Moreover, the implementation of better practices by the European Union for the transition towards zero carbon emissions by means of the development of sustainable cities is being encouraged.

In this context, biomass plays an important role as a renewable energy source. In recent years, there has been a great deal of interest in the production of bio-products starting from biomass and with different origins, thus contributing towards the transition to a circular economy [2,3]. Bio-products with the highest commercial potential are those with enhanced properties and low cost, where high added value biomass-derived carbons, also known as biochar, are included [2]. Carbon-based materials are used in a wide range of applications, including those related with energy acting as a catalyst support, material for electrode development, or gas storage [4].

Furthermore, the worldwide demand for activated carbons has increased drastically, with the market centered in the USA and China, which may lead to an increase in the price of activated carbon. Therefore, the use of activated carbons could be limited due to the high cost involved. For these reasons, research focused on the production of efficient and economic carbons from renewable and economic sources is gaining interest. Such renewable and economic sources include biomass coming from agricultural or forestry wastes, among others. In this way, it is possible to diminish the environmental impact of produced wastes and a material with high added value is also obtained [5].

In the pyrolysis of biomass, apart from the solid carbonaceous material, two more by-products are obtained: A liquid by-product named bio-oil for biofuel development and gas composed mainly of CO_2_, CO, and H_2_ from combustion of the volatile matter present in the biomass feedstock. The solid product from the pyrolysis of biomass is known as char or biochar. This material can be activated further in order to develop porosity and functionalities and the material thus obtained can be referred to as activated biochar or activated carbon. Both terms are used to designate the final product after the activation process of carbonized biomass.

Carbon-based materials have attracted the interest of scientists working on energy-related topics, such as energy production and storage; catalysis and electrocatalysis; and gas adsorption, separation, and sequestration. This is partly due to their abundance, chemical and thermal stability, and the possibility of modifying their structural features in order to fulfill the requirements of a selected application.

## 2. Sustainable Carbon-Based Materials

Methods for the synthesis of carbon-based materials need tedious procedures as well as organic solvents and electrochemical treatments. Moreover, these methods frequently depend on precursors coming from fossil fuels, procedures using metal-based catalysts, and equipment and procedures that need high temperatures for their operation [6]. None of the previous processes are environmentally and economically sustainable. These drawbacks imply an increase in production cost and limit commercialization and application in a large scale of developed carbon-based materials [7]. As an alternative, methods for the thermochemical conversion of biomass (pyrolysis and hydrothermal carbonization) have been developed. These methods give rise to low-cost carbon-based materials, require lower operation temperatures, and in addition, these processes are more environmentally friendly than those previously reported. Furthermore, precursors employed are from natural origin without the need of toxic chemical compounds [8,9,10].

### 2.1. Synthesis of Sustainable Carbon-Based Materials 

**Scheme 1:**
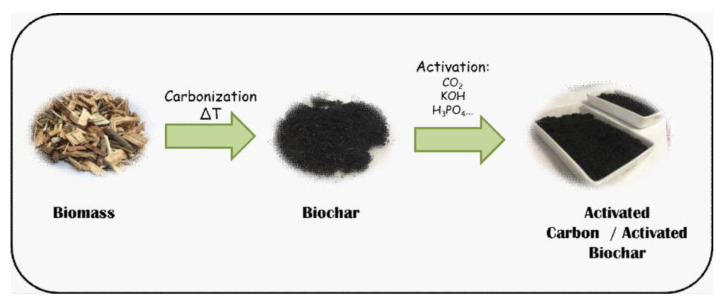
Generation of biochar-based materials: biochar and activated carbon/activated biochar from biomass.

Scheme 1 summarizes the synthetic procedure of sustainable carbon-based materials. The main product from the thermochemical conversion of biomass is biochar. Biochar is a carbon-based solid material with aromatic, aliphatic, and furanic groups together with oxygenated defects on its structure. Biochar is obtained mainly from the carbonization, pyrolysis, or gasification of biomass. In addition, biochar can also be obtained as slurry by means of hydrothermal carbonization [7]. 

Pyrolysis is the most common procedure for the synthesis of biochar [8,9,10,11]. The process for obtaining carbon derived from biomass begins with the carbonization step [12]. Carbons resulting from carbonization do not show a great development of porosity. For further development of porosity in the biomass-derived carbon (i.e., biochar), an activation process using an activating agent, such as CO_2_, acids, or bases, is followed [13]. This creates new and uniform pores that give rise to activated carbon also known as activated biochar.

### 2.2. Properties of Biomass-Derived Carbons 

An important point in the synthesis of biomass-derived carbon is related with the precursor origin and therefore its chemical composition. The most commonly used biomass in the production of biochar-based materials (i.e., biochar, activated carbon/activated biochar) usually has a lignocellulosic origin with high contents in lignin, cellulose, and hemicellulose [8,10]. In contrast, animal waste and aquatic materials with a low content in lignin and cellulose have not been evaluated with this aim in mind due to difficult operation handling [7]. The chemical composition of biomass has a great influence on the surface composition, reactivity, biochar yield, and on the properties of the final material [4,5,6,7,13,14,15,16]. These properties include the atomic ratios, elemental composition, energy content, ash content, surface reactivity, acidity, porosity, surface area, and thermal, mechanical, and chemical stability [17]. Among them, the surface area and porosity are essential properties that determine the suitability of biochar-derived materials as a catalyst support [18]. 

#### Surface Area and Porosity

A high surface area is related with other properties of the biochar-based materials, such as the cationic exchange capacity, water adsorption, or metal dispersity and loading [19], and therefore is of importance in several applications. The final textural properties of the biochar will depend predominantly on the biomass type used as raw materials during the carbonization step. With suitable conditions for carbonization, it is possible to obtain materials with several hundred m^2^/g [17]. Porosity in porous solid materials, among them biomass-derived carbon, can be classified as: Micropores (pore size < 2 nm), mesopores (2 nm < pore size < 50 nm), and macropores (pore size > 50 nm) according to IUPAC (International Union of Pure and Applied Chemistry) [19].

The porosity of samples is usually measured by gas adsorption analysis. Nitrogen adsorption at −196 °C is used for specific surface area (BET area) evaluation and pore size distribution determination. However, the limitation for the N_2_ adsorption experiment in the smallest micropore measurements should be borne in mind. A complementary experiment is the use of CO_2_ adsorption at 0 °C in order to determine the smallest micropores. In addition, H_2_ and O_2_ gases have demonstrated consistency with the results obtained for the adsorption isotherms recorded in N_2_ and CO_2_ for microporous and nanoporous carbon-based materials [20,21]. A high surface area composed of pores in the micro-scale (micropores) could not be accessible for some substrates. In the same way, pores in the macro scale (macropores) are not recommended for several applications, such as catalysis [18,22], since proximity is a desirable condition in heterogeneous catalysis [23,24]. Hence, the importance of the pore size and pore volume developed when biochar-based materials are synthesized [25,26,27].

**Figure 1:**
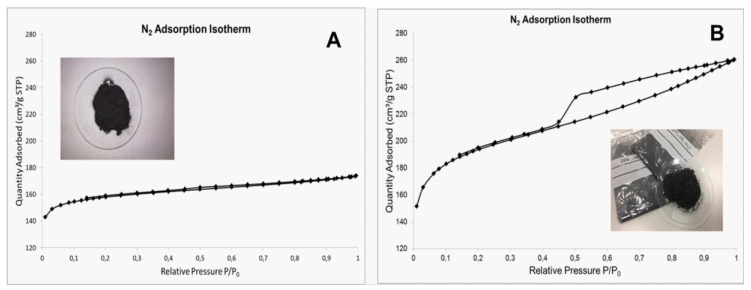
N_2_ adsorption isotherm for the activated carbon: (**A**) derived from sawdust; (**B**): derived from pruning remains.

As an example, in Figure 1, the N_2_ adsorption-desorption isotherms are presented for two different biomass-derived carbon samples treated by us under the same conditions. These materials were obtained from the pyrolysis and activation of sawdust and pruning remains. 

Figure 1 shows that the isotherms obtained differ from one other. In the case of sawdust (Figure 1a), the isotherm clearly shows a type I isotherm, corresponding to microporous materials according to the IUPAC classification [19]. In contrast, carbon material obtained from pruning (Figure 1b) has a type IV isotherm, corresponding to micro-mesoporous material with the presence of the characteristic hysteresis loop [27,28]. Different textural parameters are listed in Table 1.

From the shape of the isotherm at low pressures it can be deduced that activated biochar coming from sawdust displays a narrower micropore size distribution in comparison to the one coming from pruning. The higher value of S_ext_ observed for the carbon obtained from pruning is in accordance with the micro-mesoporous nature observed for this sample. As a conclusion, these results corroborated that the final textural properties of the carbon obtained for biomass will depend on the biomass origin.

Other properties, such as the elemental composition, ash content, and thermal stability, are of importance, especially in catalysis. [29]. In some cases, the inorganic species present may play a negative role, affecting the biochar-based material stability [17]. However, in some cases, high ash content may increase the catalytic performance [29]. Thermal stability is also of importance in high-temperature processes, such as CO_2_ methanation and other catalytic processes carried out at temperatures above 150 °C. Biomass-derived carbons have demonstrated thermal stability up to 300 °C depending on the origin of the starting material [19,30]. This is of great importance when there are organic functionalities present since functional groups must be stable under operation conditions in order to avoid thermal decomposition [19].

## 3. Biochar-Based Materials for Catalysis

Porous carbon-based materials are versatile supports for the preparation of heterogeneous catalysts [31,32,33,34]. Their surface properties can be adapted to obtain high surface areas that enhance the dispersity of active phases and tailored pore sizes that allow for proper diffusion of reagents and products [19].

Characterization of carbon derived from biomass will determine its suitability for catalysis. It is well known that the performance of a metal-based catalyst is related with the supporting material. Biochar-based materials have been studied as a supporting material to stabilize metallic nanoparticles and metal-based nanocatalysts due to their surface area and functionalities. This will be the scope of the next section focused on the metal-supported catalysts in hydrogen energy, oxygen electrocatalysis, and the reaction of CO_2_ methanation. All of them are processes involved in energy-related applications that need a catalyst to be addressed. The results obtained from the surface area and porosity analysis will be the key to establishing the best candidate to be used as support for an active metal catalyst. From the carbons evaluated in Figure 1, carbon derived from pruning remains seems to be a suitable material to be used as a catalyst support due to the presence of mesoporosity. Mesoporous materials provide a good compromise between the pore size and availability of the surface area for the reactants, since smaller pore sizes in the micro scale tend to be blocked faster. In this sense, microporous materials are useful in gas capture and the removal of pollutants [4]. 

### 3.1. Biochar-Based Materials as Catalyst Support for Energy Applications

The development of high-performance and cost-effective materials is crucial for an efficient energy storage and conversion strategy [35]. These materials should be industrially and economically attractive and synthesized from renewable and naturally abundant resources [36]. 

Carbon-based materials have played an important role in emerging energy conversion and storage technologies [37], including hydrogen storage and production [36], oxygen electrocatalysis [38], and electrodes for energy storage devices, such as supercapacitors [37] and batteries [39]. In particular, biochar-based functional materials offer many advantages due to their tunable porosity and surface area. Therefore, biochar-based materials have been widely used in the preparation of nanostructured composites that can be applied as materials for energy storage and conversion [32]. Regarding catalysis applications, biochar-based materials offer excellent physicochemical properties to be used as support for metal nanoparticles [32,40,41]. The high surface area and tunable pore structure make biochar an excellent material to be used in the dispersion of metal nanoparticles. In this way, the loading and performance of metal nanoparticles is enhanced [32]. In addition, its chemical and thermal stability allows biochar-based materials to be used in a wide range of conditions, thus conferring the suitability to be used as a support for the nanoparticle-based catalyst. Supported metal nanoparticles on biochar-based materials and other carbon-based nanomaterials have been employed in several processes, including hydrogen production and storage or the methanation reaction of carbon monoxide/dioxide, photocatalysis, and environmental remediation [41,42]. In this section, we will focus on the use of biochar as a supporting material for metal oxides and/or nanoparticles and the use of this supported catalyst in selected energy applications, such as hydrogen storage and production and the methanation reaction of carbon dioxide. 

#### 3.1.1. Biochar for Hydrogen Energy

Hydrogen is a good energy carrier due to its high energy density, availability, and abundance. Together with the high energy output, hydrogen combustion produces water as the sole final product [43]. These features make hydrogen economy one of the most ideal approaches to address fossil fuel depletion, environmental pollution, and global warming [4]. Carbon-based materials have been evaluated for hydrogen adsorption because of their high surface area, large pore volume, good chemical stability, and easy tailored porosity. However, the biochar-based material capacity for hydrogen adsorption is less than 1% *wt* at room temperature and this capacity does not increase even when high pressures are applied. Nonetheless, the hydrogen adsorption capacity can be enhanced with the use of metal-decorated porous carbon materials [4,42]. For example, Pd-decorated carbon from sepiolite exhibited a storage capacity 4 times higher than the raw material without Pd doping at room temperature [44]. In another example, Pd nanoparticles on wood-derived biochar increased hydrogen uptake due to a decrease in the activation energy of the adsorption process [45]. Nevertheless, the use of Pt and Pd nanoparticles implies high sensitivity towards air and moisture. Moreover, the embedded nanoparticles provoke a reduction in surface area and pore blocking, decreasing the adsorption capacity [46].

Another approach for H_2_ production deals with the use of a metal-free wood-derived biochar as catalyst [47,48]. This material has been used for the catalytic conversion of methane as an effective, easy to regenerate, and cheap catalyst for converting hydrocarbons (CH_4_ or tar) into syngas (CO, CO_2_, and H_2_).

For the use of biochar-based materials and supported nanoparticles on biochar-based materials for hydrogen storage, there is still research work to do in order to address specific challenges related with stability under general working conditions, and insensitivity towards moisture, air, and other gas impurities.

The high energy density of hydrogen fuel and its ability to produce energy without CO_2_ emissions make hydrogen an interesting energy source for stationary and vehicular applications [49]. The electrocatalytic hydrogen evolution reaction (HER) through water splitting is an alternative to current systems using fossil fuels. During electrocatalytic hydrogen evolution, the electrocatalyst is in charge of decreasing the overpotential and reaching high catalytic current densities. Efficient hydrogen evolution from water with a biochar-supported catalyst appears as an outstanding alternative to produce clean and renewable hydrogen as a fuel. The incorporation of active species on the surface of biochar-based materials is an effective route towards the development of high-performance HER electrocatalysts [50,51,52,53,54]. Catalysts capable of performing HER in acidic media are desirable since in this way, there is compatibility with the existing proton exchange membrane technologies; they exhibit lower ohmic loss and gas crossover is reduced [55]. However, this reaction has been carried out for many years using a precious metal (Pt, Ru) electrocatalyst. The use of such precious metals implies a high cost for catalyst development due to the scarcity of these materials. To address this issue, other compounds, such as transition metal chalcogenides [53,56], phosphides [50,52,57,58], nitrides [59], and carbides [60,61,62], among others, have emerged as less expensive alternatives for electrocatalytic HER. Other transition metal-based materials, such as cobalt [50], nickel [63], iron [64], or copper [65] phosphides, have been employed as an active phase in HER catalysts without the use of a support. Overpotentials and current densities for electrocatalytic HER using a transition metal-based catalyst are within the range from 25 to 150 mV and 10 to 100 mA/cm^2^ respectively. For example, NiP exhibits overpotentials of about 25–50 mV with current densities of 20–100 mA/cm^2^ [63]. The problem with an Ni-based electrocatalyst arises from its low stability in acidic media [63,65]. In the case of an iron-based catalyst, they have attracted the interest of researchers due to their abundance and low cost compared with Co and Ni [64]. Overpotential for an FeP-based catalyst is about 50 mV for a current density of 10 mA/cm^2^. Copper-based electrocatalysts have also been employed in HER due to their low cost. These catalysts showed higher overpotential in regard to Ni-, Co-, and Fe-based electrocatalysts. The overpotential recorded for Cu_3_P was about 143 mV for a current density of 10 mA/cm^2^. The main challenges to be addressed for copper-based electrocatalysts are to decrease the overpotential and increase the long term stability. 

**Figure 2:**
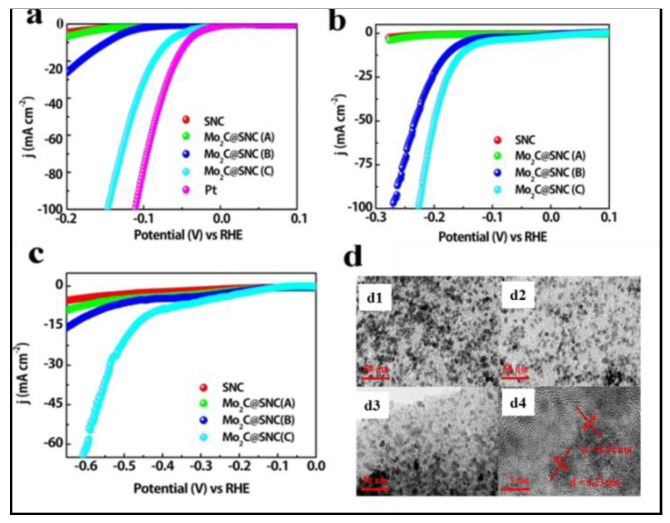
Polarization curves of sulphur-nitrogen-doped carbon (SNC) and Mo_2_C@SNC (**a**) in 1 M KOH; (**b**) in 0.5 M H_2_SO_4_; (**c**) in a neutral PB (Phosphate Buffer) solution; (**d1**–**d3**) TEM image of Mo_2_C@SNC catalyst prepared using different Mo precursor concentrations; (**d4)** high-resolution TEM image of one of the Mo_2_C@SNC catalysts. Reproduced from [67] with permission of the American Chemical Society 2020.

One of the compounds that has received more attention due to its electronic structure being similar to that of Pt is molybdene carbide (Mo_x_C). In addition, Mo_x_C compounds have a low cost and show chemical stability [62]. Moreover, these materials have demonstrated excellent catalytic activity for the HER when coupled with carbon-based materials (Figure 2) due to an increase in the exposed surface area and to an enhancement of electron transport ability [66]. However, the use of these last-generation carbon-based materials increases the total cost of the catalyst.

**Figure 3:**
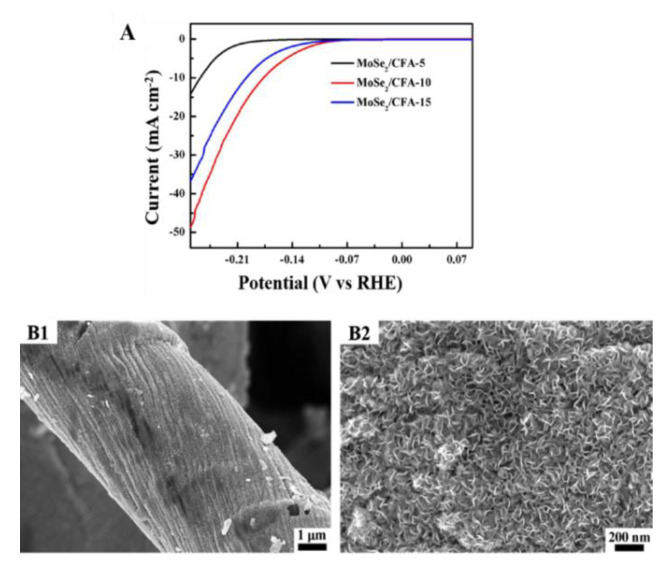
(**A**): Linear Sweep Voltammetry (LSV) polarization curves for MoSe_2_/CFA (Carbon Fiber Aerogel) hybrid modified Glassy Carbon Electrode GCE. (**B**): FESEM images of MoSe2/CFA hybrid (**B1,B2**). Reproduced from [68] with permission of the American Chemical Society 2020.

Biochar-based materials have also found application as a catalyst support in HER. For this purpose, Mo nanoparticles have been incorporated in biochar to give rise to a biochar-nanostructured composite with activity for electrocatalytic HER. Mo_2_C nanoparticles have been grown on biomass in the form of soybeans as a low-cost carbon support with an overpotential of 177 mV to drive a current density of 10 mA/cm^2^ [54]. In particular, biochar derived from sunflower seed shell biomass was modified with Mo_2_C nanoparticles [67]. This nanoparticle-based catalyst needed an overpotential of 60 mV to deliver a current density of 10 mA/cm^2^ as shown in Figure 3. This catalyst displayed good stability and a faradaic efficiency of about 100%.

Carbon fiber derived from cotton wool biomass has also been used as the supporting material for MoSe_2_ nanosheets. The material showed excellent catalytic activity for HER with a small onset potential of 104 mV as shown in Figure 3 [68]. In recent years, the development of cheap Mo-based Pt-free HER electrocatalysts with high activity has been extended and is one of the most studied transition metals for HER due to its abundance and low cost. Furthermore, MoP has demonstrated good catalytic activity for various hydroprocessing reactions [52] and this behavior has been extrapolated to electrocatalytic HER.

Despite the use of biochar-based materials as catalyst support for HER, there are still some challenges that need to be addressed. These challenges are related with elucidation of the catalytic mechanism of biochar-based materials in electrocatalytic HER, identification of catalytic active sites, and an increase in the number of such catalytic active sites. Besides, more advanced synthetic methods to prepare biochar-based materials for HER with good atomic dispersion of the doping elements should be developed [4].

#### 3.1.2. Biochar for Oxygen Electrocatalysis

The oxygen reduction reaction (ORR) and the oxygen evolution reaction (OER) are two of the most important reactions taking place in energy storage and conversion systems, such as metal-air batteries and fuel cells. [69]. The efficiency of these reactions is restricted by sluggish reaction kinetics and therefore the use of a catalyst is needed to overcome the energetic barrier. Conventional ORR and OER catalysts are based on noble metals and their oxides (for example, Pt, Pd, RuO_2_, IrO_2_). However, their poor stability under working conditions as well as their high cost and scarcity limit their use in large-scale applications [4].

The most widely used catalysts for ORR are based on Pt and Pt alloys. However, their low availability and high cost hinder their use in fuel cells and metal-air batteries. In addition, a Pt-based catalyst suffers from low long-term stability and low tolerance to methanol and CO [70]. Therefore, the challenge relies on the development of alternative ORR catalysts, with low cost, based on earth-abundant components, with high catalytic activity, operation stability, and tolerance towards methanol and CO [71]. Biochar-based materials can afford a suitable supporting matrix with high surface area and catalytic active sites bearing heteroatoms or active metal compounds.

The incorporation of low-cost and abundant transition metals, such as Fe, Co, and Ni, in biomass-derived carbon doped with heteroatoms is an effective way to obtain a catalyst with good performance for ORR [72,73,74]. For example, nitrogen-doped biochar, from soybean pyrolysis that has been modified with Fe, leads to the appearance of active sites with enhanced performance for ORR [75]. Furthermore, nitrogen-doped biochar used as a catalyst support for ORR improves catalytic activity and durability due to the formation of π-bonds and the remarkable electron donor behavior of nitrogen. Pd nanoparticles supported on this N-doped carbon from biomass pyrolysis show remarkable electrochemical activity for ORR with an increase in catalytic performance and long-term stability [76].

On the other hand, OER takes place in various technologies of energy conversion and storage, such as water splitting or rechargeable metal-air batteries. This reaction involves a process with four electrons, which leads to slow reaction kinetics. Therefore, the presence of a catalyst is necessary in order to increase the reaction rate and to decrease the overpotential [4]. Oxides from noble metal, such as IrO_2_ and RuO_2_, are the most commonly used catalyst for OER. These catalysts show high catalytic activity and stability, but like for the case of ORR and H_2_ production, their high cost and scarcity hinder their commercialization. Therefore, the development of alternative catalysts for OER is a key step in energy conversion and storage technologies.

**Figure 4:**
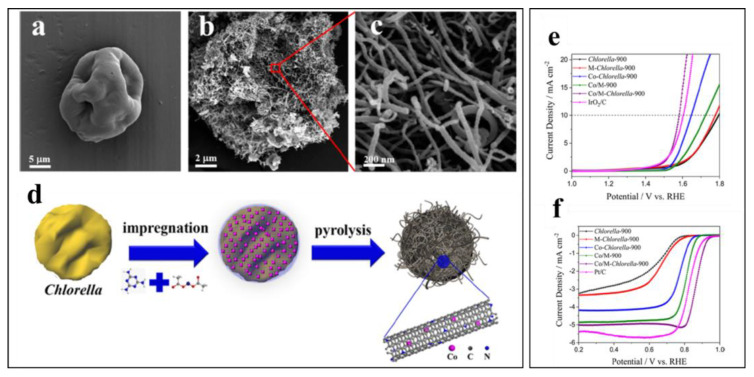
(**a**) SEM image of *Chlorella* algae and (**b**), (**c**) SEM images of Co/M-Chlorella-900. (**d**) schematic illustration of the preparation process of Co/M-Chlorella-900. (**e**) LSV curves for five catalysts from *Chlorella* algae and 20 *wt* % commercial Pt/C in 0.1 M KOH solution. (**f**) LSV curves for five catalysts from *Chlorella* algae and commercial IrO_2_/C in 0.1 M KOH solution. Reproduced from [77] with permission of the American Chemical Society 2020.

Biochar, due to its abundance in nature, porous structure, and tunable surface chemistry, has emerged as an ideal support for the dispersion of active metals for OER. One of the most recent examples of metal nanoparticles supported on carbon derived from biomass pyrolysis was reported by Wang and co-workers [77]. Co nanoparticles were dispersed by impregnation in biomass derived from *Chlorella* algae. After pyrolysis at 900 °C during 1 h under Ar atmosphere, a carbon nanotube-like material was obtained with Co nanoparticles in its structure (Figure 4). These nanoparticles were able to act as a bifunctional catalyst for both ORR and OER. For OER, the overpotential at 10 mA/cm^2^ was 23 mV lower than the overpotential recorded for a reference catalyst of IrO_2_/C (Figure 4e). For ORR, the half-wave potential was 40 mV higher than that obtained for a reference catalyst based on 20% wt Pt/C (Figure 4f). As far as we are aware, no further metal-nanoparticles supported on biomass-derived carbon materials have been reported as an active catalyst for OER.

#### 3.1.3. Biochar for Methanation of Carbon Dioxide

The Summit for Climate Action held on September 2019 announced that 77 countries, 10 regions, and 100 cities were all committed to reaching net zero carbon emissions by the year 2050 [78]. Advances in the development of technologies with zero carbon emissions will help to accomplish the stated objectives. In this sense, carbon capture, utilization, and storage (CCUS) technologies are of great interest, especially considering that fossil fuels will continue playing an important role in the global energy supply during the transition. However, CCUS technologies are still expensive and need further development for their real application. In contrast, renewable energies play a critical role in the transition towards a sustainable energy system [44]. Taking this into account, the conversion of renewable energy in H_2_ offers the possibility of storing chemical energy. However, due to the intrinsic low density of H_2_ molecules, H_2_ storage and transport constitute a great challenge. As an alternative, the conversion of CO_2_ into methane is an important approach for the transformation of energy from renewable sources. In addition, it is expected that the market of methane utilization will grow between 4 and 65 billion cubic meters per year by 2030 [78].

**Scheme 2:**
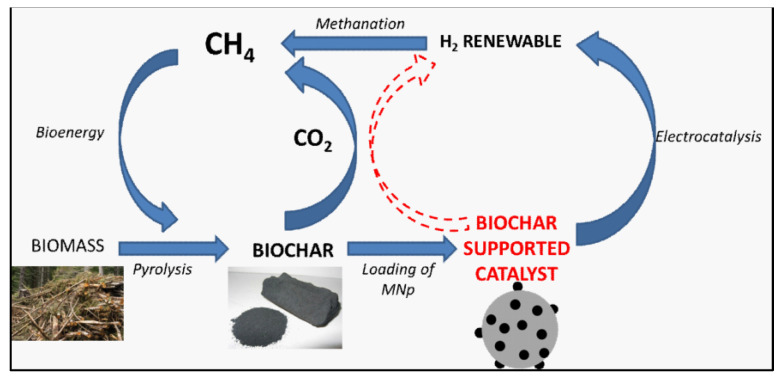
Integration of renewable energies with the C cycle to produce fuels and reduce CO_2_ emissions.

Among the different fossil fuels, natural gas, composed mainly of methane, is ideal due to its high energy density and efficiency, ease of transport due to the existing infrastructure, and composition free of slag [79]. The increase in the price of natural gas along with the wish to diminish dependence on importation has boosted the production of synthetic natural gas (SNG) from renewable biomass, coke gas, or syn gas from the wood and coal industry [80]. Besides, electrocatalytic or photocatalytic hydrogen production using renewable energy sources is considered to be one of the most promising hydrogen sources for CO_2_ methanation. Thus, the production of SNG via CO_2_ methanation not only produces a fuel, such as natural gas, it also contributes to the reduction of greenhouse gas emissions into the atmosphere as shown in Scheme 2 [80]. In addition, methanation can also be used to reduce CO traces in hydrogen production feedstock gases in order to obtain high-purity hydrogen, for example, for the chemical industry, fuel cells, or methanation reaction.

There are different ways to transform CO_2_ in methane, including photosynthesis and photocatalysis, electrochemical CO_2_ reduction, CO_2_ biological conversion, and thermo catalytic CO_2_ methanation [81,82,83,84,85]. Recent studies have considered the potential of power to gas (PtG) technologies (thermo catalytic CO_2_ methanation) for the future generation of energy [81,84]. With this technology, electric energy from renewable sources is transformed into methane by electrolysis and catalytic methanation. The methane thus produced can be stored and transformed using the existing infrastructure for natural gas in a way similar to that presented in Scheme 2. This would help in long-term decarbonization and reduce global warming due to the reutilization of CO_2_ instead of being released into the atmosphere [78]. The CO_2_ used will come from biogas, natural gas, pure CO_2_, or from exhausted gases. However, the main challenge of this approach relies on the high cost of methane production by thermo catalytic methanation when compared with conventional methods [44]. This implies that there is a need to improve efficiency and to reduce the cost of the process. This situation has led to an increase in research work focused on CO_2_ methanation. The two reactions involved in the methanation can be expressed as:

CO_2_ + 4H_2_ = > CH_4_ + 2H_2_O, (1)

CO + 3H_2_ = > CH_4_ + H_2_O. (2)

Reaction (1) deals with the methanation of CO_2_ while Reaction (2) deals with the methanation of CO. Methanation of CO is mainly applied for the production of SNG from gas streams with high concentrations of CO and/or CO_2_ [80]. In addition, CO methanation can also be employed in the removal of trace amounts of CO from H_2_ streams [86]. Meanwhile, methanation of CO_2_ has gained attention in recent years as a way for CO_2_ capture and reutilization, diminishing greenhouse gas emissions. Although both reactions are favorable thermodynamically, a catalyst is needed in order to have an appropriate reaction rate. CO_2_ transformation at high temperatures (> 450 °C) gives rise to the water gas-shift reaction that generates CO (Reaction 3) and reduces selectivity towards CH_4_ [80]:

CO_2_ + H_2_ = > CO + H_2_O. (3)

**Figure 5:**
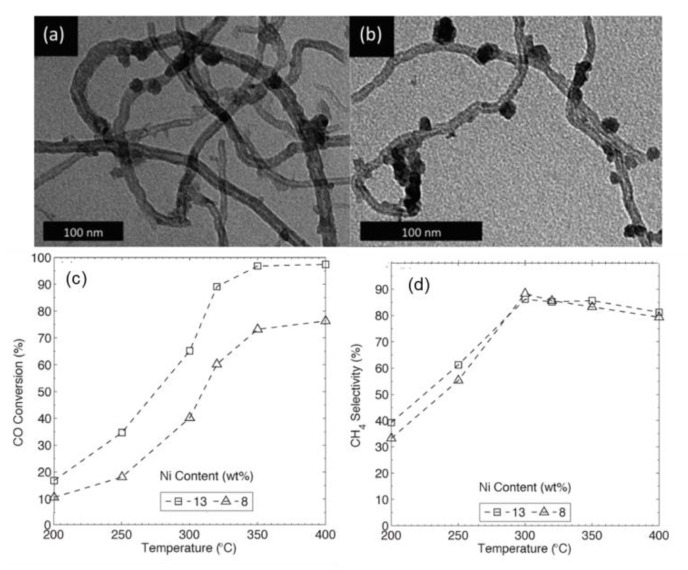
Ni nanoparticles supported on carbon nanotubes (CNTs) as catalyst for the methanation reaction (**a**,**b**). CO conversion and CH_4_ selectivity, (**c**,**d**), for selected Ni-CNT catalysts. Reproduced from [86] with permission of The Royal Society of Chemistry 2020.

Therefore, it is desirable to carry out the methanation reaction at a low temperature (<300 °C). However, at this temperature, it is difficult to reach a suitable reaction rate and selectivity requiring, then, the presence of a highly active catalyst able to overcome the energetic barrier. Sabatier and Senderens found in 1902 that Ni showed good catalytic activity for the methanation reaction of CO_2_. Since then, numerous studies have been carried out and among them, the work of Vannice and co-workers evaluated the use of metals of the group VII (Fe, Co, Ni, Ru, Rh, Pd, Ir, Pt) for hydrocarbon production starting from H_2_ and CO. Methanation catalysts are composed of metal particles dispersed on the surface of a support usually based on metal oxides, such as alumina or silica [80]. Support materials play an important role in heterogeneous catalysis manly due to the enhancement of catalytic activity by means of catalyst–metal dispersion, metal–support interactions, electron transfer between the metal and the support, and the presence of defect sites [78]. Many different types of supporting materials have been reported for methanation catalyst, including Al_2_O_3_, SiO_2_, ZrO_2_, TiO_2_, and CeO_2_; as well as structured metal oxides, carbon, and zeolite materials [78,80,85]. Among the carbon materials that have been employed as a support for methanation catalysts, we found carbon nanotubes [86,87], activated carbon [88], carbon nanofiber [89], biochar-based materials [90,91,92], and carbon felt [93]. As an example, carbon nanotubes have been widely used as a support of metal nanoparticles due to their exceptional physico-chemical properties. Ni nanoparticles supported on carbon nanotubes have demonstrated a remarkable activity for the methanation reaction as shown in Figure 5 [86]. The use of carbon-based materials as a support offers numerous benefits, such as, for example, the presence of adsorption sites, conductivity and thermal stability to manage temperature gradients, tunable surface properties, high surface area, and reduced tendency to coke deposition that leads to catalyst deactivation [4,32]. In recent decades, carbon-based materials have been widely used as a support in catalysis [18,32,94,95]. However, the use of biochar, the product of the thermal conversion of biomass, has not been explored deeply and is mainly discarded or burnt as a fuel.

The properties of biochar are similar to those of commercial-activated carbon: High surface areas and surface chemistry that allow its modification. Moreover, active compounds can be recovered using oxidation methods [78]. Therefore, biochar is a renewable source of carbon that can be obtained at a low cost with a high potential to be used as a catalyst support. For instance, biochar has been employed as a support for iron nanoparticles in Fisher–Tropsch synthesis for bio-syngas [96]. 

Nevertheless, there are very few reports on the use of biochar as a catalyst support for the methanation reaction of carbon dioxide. Most of these studies deal with the methanation of syngas. This gas usually consists of H_2_, CO, and CO_2_, with CO being the main component. The idea is to perform the methanation reaction over this syngas coming from combustion facing the challenges related with environmental sustainability and a circular economy due to the reuse of exhausted gases at the same time as greenhouse gas emissions are minimized (Scheme 2). In one of these studies, Wang et al. used a biochar derived from Lauan (a kind of tree) that was activated in order to develop a pore structure that allowed its use as a catalyst support [90]. The activated biochar showed a BET area of 1100 m^2^/g and its stability was similar to that obtained for a commercial-activated carbon. Ru nanoparticles were incorporated onto the activated biochar surface by impregnation and three catalysts with different Ru loadings were obtained. The catalytic activity for the methanation reaction of a simulated bio-syngas (H_2_, CO, CO_2_ from biomass combustion) was evaluated in a continuous flow fixed-bed reactor and the influence of parameters, such as the H_2_/C ratio or temperature, on catalytic performance were studied. Ru on activated biochar (ABC) was able to perform a methanation reaction of a simulated bio-syngas with a CO conversion of 97% at 420 °C under a H_2_/C ratio of 4. CH_4_ selectivity and yield were 92% and 54%, respectively. In addition, the supported Ru catalyst showed better dispersity than that found with Al_2_O_3_ or SiO_2_ supports with the same Ru loading. 

In another study by the same group, rice husk was pyrolyzed and activated in order to obtain an activated biochar to be used as a support for an Ru-based methanation catalyst [91]. Moreover, an Ru catalyst over conventional activated carbon was also prepared for comparison. This time, the syngas methanation reaction proceeded at a temperature of 340 °C. CO conversion and CH_4_ selectivity for Ru catalyst on ABC were 100% and 98%, respectively. In the case of Ru catalyst over conventional activated carbon, CO conversion at 360 °C was 99% while selectivity was about 85%. Moreover, Ru supported on ABC showed stability for a period of 100 h on stream without decreasing conversion and selectivity.

**Figure 6:**
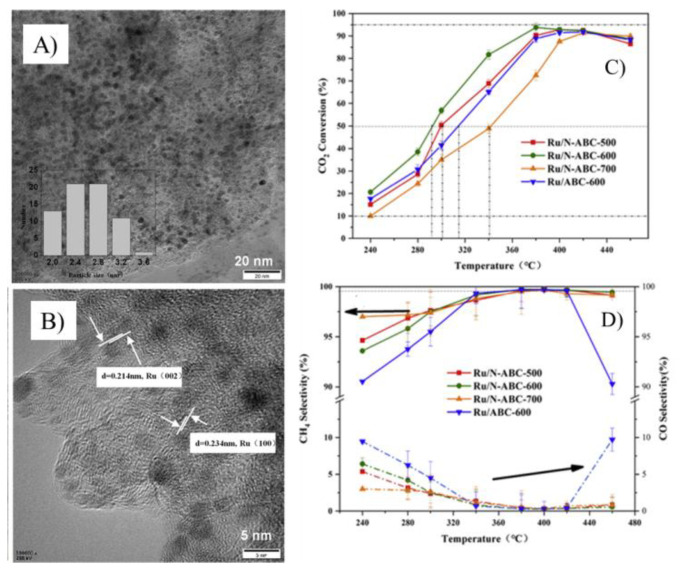
(**A**) TEM and HRTEM (**B**) images of the catalysts Ru/N-ABC pyrolyzed at 600 °C; the inset in part (**A**) displays the particle size distribution obtained by TEM. Temperature dependence of CO_2_ methanation over the catalysts: (**C**) the CO_2_ conversion; (**D**) the CH_4_ and CO selectivity. Reproduced from [92] with permission from Elsevier 2020.

For CO_2_ methanation, an N-doped biochar from *Pynus sylvestris* was obtained by pyrolysis and activation using urea as the nitrogen precursor and NaHCO_3_ as the activating agent [92]. Biochar obtained in this way was modified with Ru nanoparticles by the wet impregnation method. The Ru-supported catalyst showed good dispersity (Figure 6a,b) and demonstrated good catalytic activity for CO_2_ methanation. N-doped biochar was obtained by pyrolysis at three different temperatures and the surface properties of the Ru-supported catalyst prepared were determined. The best catalytic material was the one obtained from the N-doped biochar pyrolyzed at 600 °C. This Ru catalyst on biochar displayed a CO_2_ conversion and selectivity of 94% and 99.7%, respectively, at 380 °C (Figure 6c,d). Moreover, the same catalyst without N doping displayed the worst catalytic performance. The authors stated that the presence of pyridinic N, the high surface/active metal component ratio, and the surface alkalinity together with the high surface area are the reasons behind the better catalytic performance of the Ru supported on N-doped biochar catalyst.

## 4. Conclusions and Future Perspectives

Biomass has demonstrated that it serves as a promising raw material for the development of sustainable carbon-based materials and added value porous carbon materials. Its properties, especially the porosity of carbon materials, can be tailored by choosing the appropriate biomass feedstock and adjusting process parameters during carbonization and the activation process. This fact, together with the chemical stability, easy modification of the surface chemistry, possible metal modification, and low-cost and sustainability, make these materials very promising for their use as a support in different catalytic processes, such as in hydrogen production or air-electrode in batteries and fuel cells, as well as for CO_2_ methanation.

Biochar-based materials as a catalyst support for hydrogen and oxygen reactions have demonstrated a great potential due to the possibility of decreasing the cost for catalyst development and increasing efficiency. The high surface area of biochar-derived materials allows for a better dispersity of the active metal on the support, minimizing the amount of metal required and increasing the catalytic performance.

Activated biochar has also demonstrated good catalytic activity for the methanation of CO_2_ using Ru as the active component. The presence of doping elements, such as N, improves the metal dispersity and surface alkalinity. This, together with the high surface area of activated biochar, results in high-performance catalytic material.

## Figures and Tables

**Table 1:** Textural properties of activated carbons obtained from the different types of biomass.

Sample	Type of Isotherm (IUPAC)	BET Analysis	t-plot Analysis		
		S_BET_ (m^2^/g) *Experimental*	Vmic (cm^3^/g)	Smic (m^2^/g)	Sext (m^2^/g)
**Carbon Derived from Pruning Remains**	Type IV (micro-mesoporous)	639	0.19	399	239
**Carbon Derived from Sawdust**	Type I (microporous)	508	0.21	433	75
